# Mating from a female perspective: Do brown bear females play an active role in mate searching?

**DOI:** 10.1186/s40462-025-00553-6

**Published:** 2025-04-03

**Authors:** Vincenzo Penteriani, María del Mar Delgado, Ilpo Kojola, Samuli Heikkinen, Ancuta Fedorca, Pino García-Sánchez, Mihai Fedorca, Slavomír Find’o, Michaela Skuban, Javier Balbontín, Alejandra Zarzo-Arias, Daniele Falcinelli, Andrés Ordiz, Jon E. Swenson

**Affiliations:** 1 Department of Evolutionary Ecology, National Museum of Natural Sciences (MNCN), Spanish National Research Council (CSIC), Madrid, Spain; 2 Biodiversity Research Institute (IMIB, CSIC-Oviedo University-Principality of Asturias), Mieres Campus, 33600 Mieres, Spain; 3 LUKE, Natural Resources Institute Finland, Ounasjoentie 6, Rovaniemi, Finland; 4 Department of Wildlife, National Institute for Research and Development in Forestry Marin Dracea, Closca Street 13, 500040 Brasov, Romania; 5 Department of Silviculture, Transilvania University of Brasov, Beethoven Line 1, Brasov, Romania; 6 School of Animal, Rural and Environmental Sciences, Nottingham Trent University, Brackenhurst Campus, Southwell, Nottinghamshire, NG25 0QF UK; 7 Carpathian Wildlife Society, Námestie Slobody 18, 960 01 Zvolen, Slovakia; 8 Departamento de Zoología, Facultad de Biología, Universidad de Sevilla, Edificio Verde, Avda. de Reina Mercedes s/n, 41012 Seville, Spain; 9 Departamento de Biología, Facultad de Biología, Universidad Autónoma de Madrid, 28049 Madrid, Spain; 10 Universidad de Oviedo, 33003 Oviedo, Asturias Spain; 11 Department of Environmental Biology (DBA), Sapienza University of Rome, 5 Piazzale Aldo Moro, 00185 Rome, Italy; 12 Departamento de Biodiversidad y Gestión Ambiental, Área de Zoología, Universidad de León, León, Spain; 13 Faculty of Environmental Sciences and Natural Resource Management, Norwegian University of Life Sciences, Ås, Norway

**Keywords:** Females, Infanticide, Mating excursions, Mating strategies, Movement ecology, Roaming–to–mate, *Ursus arctos*

## Abstract

**Background:**

Limited information exists on the active role of females during mate searching. Theory primarily focuses on male reproductive behaviours, suggesting male distribution follows that of females, while female distribution is influenced by food resources and habitat. This approach might underestimate the females’ role in shaping mating strategies. Incorporating a female perspective into mating studies can enhance our understanding of evolutionary factors.

**Methods:**

Using GPS data from brown bears *Ursus arctos* across Finland, Romania and Slovakia, we explored female movement behaviour during the mating period. First, we estimated movement speed, total distance and net distance at a daily scale. Then, we quantitatively described when the movement peaks occur by estimating two critical points of the functions described by each of the aforementioned movement parameters: (1) the point in time when the rate of change in brown bear movement behaviour is the highest; and (2) the point in time when each aspect of brown bear movement is most pronounced. We quantified temporal variations in male and female movements throughout the year using generalized additive mixed models, while we used linear mixed models to assess the relationship between peak movement parameters, bear sex and population.

**Results:**

Our findings identified two overlooked behaviours: (1) male and female movement parameters showed the highest rate of change during the mating season, challenging the notion of male roaming as the primary mating strategy; and (2) females travelled the longest distances during the mating season, potentially seeking high-quality mates. This behaviour aligns with the strategy of engaging in copulations with multiple males to avoid infanticide.

**Conclusions:**

Our study reveals novel insights into the active role of female brown bears in mating strategies, challenging traditional male-centric views. These results support the need for detailed investigations into female behaviours across mammalian taxa, which offer potential to advance our understanding of mammalian social and mating systems. Local differences also underscore the importance of social and ecological conditions to explain variation in the female role in mating strategies.

**Supplementary Information:**

The online version contains supplementary material available at 10.1186/s40462-025-00553-6.

## Introduction

In recent decades, there has been increasing interest in understanding mating system dynamics and strategies from the female perspective across various taxa, including mammals, birds, fish and amphibians [1–6]. Although considerable attention has been dedicated to female mate choice in mammals [1, 4, 6, 7], there is still limited information available regarding the spatial components of the active role of females during mate searching. Despite potential explanations for female mammal mating tactics being derived from direct benefits resulting from female preferences [6, 7], studies typically focus on male reproductive behaviours and strategies, as males generally exhibit more variation in reproductive success than females [4, 8]. As an example, when spatial associations between the sexes are recorded during the mating season, the common male-focused perception and conclusions are often that males are in the same location as females, and not vice versa [6, 8, 9]. This aligns with the idea that males move to areas where females occur, with no mention of what the females do. A widespread viewpoint suggests that male distribution largely follows that of females, whereas female distribution during the mating season is primarily determined by food resources and optimal habitat [10–12]. However, this approach might underestimate the role of females in shaping the mating system of a species [6].

Incorporating a female perspective in studies on mating may enhance our understanding of factors that influence the evolution of different mating strategies. For example, recent developments in sexual selection research have been driven by a more detailed exploration of female influences on mate choice and increased emphasis on interactions between the sexes [1, 3]. Specifically, it is now recognised that females across diverse animal taxa typically mate with more than one male per mating season [13, 14], with potentially significant implications for the evolution of both male and female reproductive strategies [3].

Scramble competition, which involves competitive searching for mates, has traditionally been viewed as a complementary or alternative male mating strategy to contest competition, particularly when receptive females are spaced widely and dispersed unpredictably [9, 15]. From the perspective of scramble competition in solitary species, spatial associations between males and females are often attributed to males moving towards areas where females are located [9]. This scenario seems to play a major role in those animal societies where males do not offer paternal assistance and females are both spatially dispersed and reproductively synchronised, making them challenging to monopolise [9]. However, despite being frequently overlooked in the past, evidence exists for female mammals competing for access to mates [3]. Mate searching has thus been recognised as a powerful driver of changes in animal behaviour during the reproductive season. One such behavioural change is the alteration of movement patterns of individuals during mate searching, such as roaming to find receptive mates, as documented in many species [16–19].

Brown bears *Ursus arctos* are typically considered as a solitary, non-territorial species, with promiscuous adult females exerting some control over mating acts and partner choice [20–22]. During a breeding season, which generally occurs between early spring and early summer [21, 22], females typically mate with multiple males [23] and have been observed initiating mating on occasions [23, 24]. The apparent success of larger, older or more aggressive male brown bears may partly be explained by female choice for these traits as indicators of genetic quality [25]. The spatiotemporal distribution of receptive females is a critical factor shaping the mating system of animal species [4, 26–29], which explains why brown bear males range widely in search of estrous females during the mating season [20, 23]. It is frequently assumed that, whereas male reproductive success is primarily limited by access to females [23], female success is generally limited by access to resources [30]. However, home ranges of brown bears are larger for both males and estrous females during the mating season than later on in the year [31, 32], likely to increase mating opportunities. Furthermore, specific female movement patterns during and after the mating season, coupled with multi-male copulations, are recognised as a strategy to reduce the risk of infanticide of cubs the year after the mating season [30, 33]. Given the necessity to mate with multiple males to exploit male uncertainty of paternity and thus decrease the risk of infanticide, actively searching for mates rather than passively waiting for roaming males might be one of the most effective female strategies. Additionally, during the mating season, adult female brown bears also exhibit an active role in chemically marking their presence on rubbing trees [34]. These findings suggest that females employ their own reproductive tactics to maximise fitness, both in terms of finding high-quality males and reducing cub mortality, suggesting a more active role in mate searching than previously assumed.

Utilising GPS data collected from brown bears of two European populations (Karelian and Carpathian) in three countries (Finland, Romania and Slovakia), we aim to move a step forward in our understanding of adult female mating behaviour by using the tools offered by movement ecology. We explored two key questions: (1) when do adult female and male brown bears experience their peak in movement parameters, characterised by the highest rate of change and maximum values in movement parameters, within the year?; and (2) are adult male and female movement peaks an intrinsic trait of the brown bear or are they also influenced by local conditions [31]? In the context of mammalian mating strategies, particularly the encounter theory [35], which suggests that increasing daily displacements improves the chance of encountering receptive mates, we hypothesise that adult females may exhibit active movement behaviours during the mating season, potentially leading to increased encounters with different mates.

## Methods

### Radio-tracking procedures

Between 2002 to 2021, 66 adult brown bears (28 males and 38 females) from three brown bear European areas (i.e., Finnish and Russian Karelia, n = 28 (11 males and 17 females); eastern and southern parts of the Romanian Carpathians, n = 19 (5 males and 14 females); north-central Slovakia, n = 18 (13 males and 7 females), were captured and equipped with GPS collars that monitored their movements from spring until they entered the winter den (mean Day of the Year (DOY) ± SD = 200 ± 26,12; range = 91–319; see also [33]. Brown bears data were therefore collected during the mating and the hyperphagia periods. In cases where data from both periods were unavailable for some individuals, we sub-sample the dataset by selecting only the bears for which data from both periods were available. After re-running the analyses, the results remained consistent (not shown here). Based on the general life cycle of this species [21, 22], the mating season (i.e., when individuals focus on reproductive activities) typically occurs from late spring to early summer (around Day of Year 91–212), whereas the hyperphagia period (i.e., when the primary focus shifts to intensive feeding in preparation for hibernation) occurs in late summer through autumn (Day of the Year 213–318). Given that these periods can vary across geographic regions and even among individuals due to temporal changes in environmental conditions, we did not pre-define these periods. Instead, we examined intra-annual variation in movement behaviour to avoid categorising behaviours a priori. Upon capture, either from blinds at temporary bait sites (Finland) or using culvert traps (Romania and Slovakia), we determined the sex of each bear, weighed them, and classified them as adults if they were older than 5 years [36–38]. Due to variations in bear physiology and body fat levels, sedative doses were adjusted seasonally. The drug dosages included a mixture of medetomidine (50 mg/kg) and ketamine (2 mg/kg), tailored to the bear’s size [39]. In late summer and early autumn, dosages were increased by 25 to 50%, and longer needles were used to accommodate higher body fat levels [40].

Finnish bears were fitted with GPS collars (Televit, Lindesberg, Sweden; Vectronic Aerospace, Berlin, Germany; for more information, see [41]). Romanian bears were equipped with GPS-GSM collars (Vectronic Aerospace, Berlin, Germany), and Slovakian bears were fitted with GPS-GSM collars (Vectronic Aerospace, Berlin, Germany) [33]. The weight of the collars represented from 0.2% to 2% of the body weight of adult bears. No negative effects were observed during bear captures and tracking procedures.

The GPS collars were calibrated to continuously track brown bears, and for the purpose of this study, we used locations collected every 2 h (see [42] for more details). This allowed collecting a total of 69,476 locations, distributed thusly among countries: 13,655 locations for Finland (males = 5,911 locations; females = 7,744 locations), 13,382 for Romania (males = 3,782 locations; females = 9,600 locations) and 42,439 for Slovakia (males = 25,380 locations; females = 17,059 locations). Signals from the satellite collars were recorded by the ARGOS satellite system (http://www.cls.fr). We recorded the positional dilution of precision (PDOP) value for all 3-D fixes and the horizontal dilution of precision for 2-D fixes. Following the method developed by [43], we excluded all 2-D fixes (8% of the locations), thus removing large location errors [44].

### Movement path analyses and critical points

First, to study the dynamics of male and female brown bear movement behaviour throughout the year, we estimated the following three movement parameters at a daily scale: (1) movement speed (*v*), dividing the step distance by the time interval between successive locations; (2) the total distance (*tot*), based on the gross distance travelled by each individual each day; and (3) the net distance (*net*) between the initial position and final position each day. Total distance reflects the actual path taken by an individual, offering a detailed understanding of how much individuals move on a finer scale. In contrast, net distance represents how far individuals move away from a specific point. Together, these two metrics provide complementary information on animal movement patterns.

**Fig. 1:**
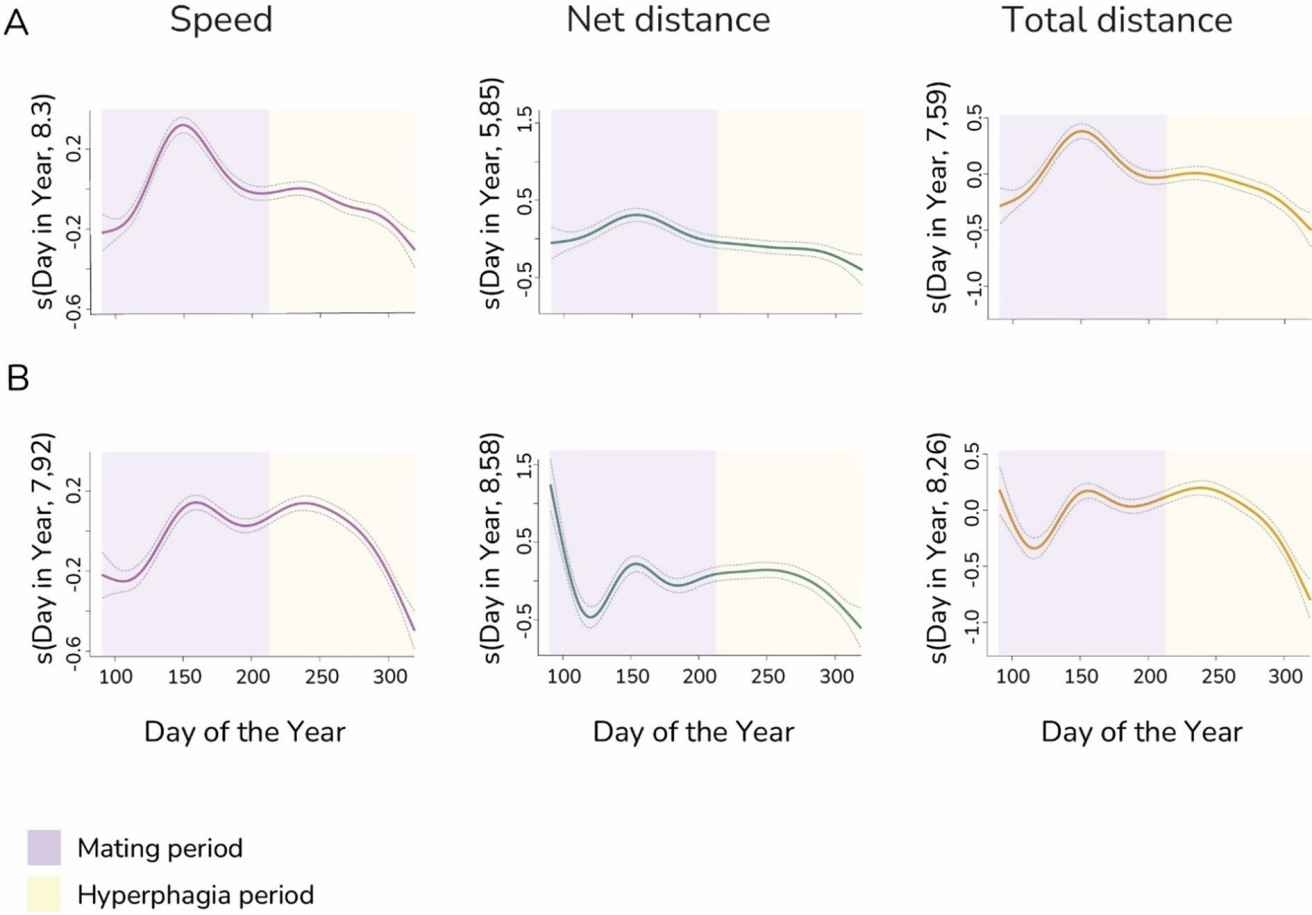
Effect of the smooth term DOY (s(Day of the Year)) for each level of sex (**A**: males; **B**: females), after accounting for the random effect of year, on brown bear speed (m/s), net distance (m) and total distance (m). The solid lines represent the predicted variation of each response variable with DOY, while the dashed lines indicate the confidence intervals. Since the response variables were modelled using a Gamma family with a log link, the estimates on the *Y*-axis are on a log scale. Data were collected in three European areas (Finnish and Russian Karelia, eastern and southern parts of the Romanian Carpathians, and north-central Slovakia) from 2002 to 2021. The transition date between the mating period, which typically occurs from late spring to early summer (DOY 91–212), and the hyperphagia period, which occurs in late summer and autumn (DOY 213–318), is based on the general life cycle of brown bears [21, 22]

Second, we quantitatively described when the peaks in brown bear movement behaviours occur by estimating two critical points in time of the functions described by each of the aforementioned movement parameters, i.e. $f\left(v\right)$, $f\left(net\right),$ and $f\left(total\right)$ (Fig. 1): (1) the day when the maximum of the first derivative of each movement parameter takes place (*k*; for a graphical representation, see Fig. 1B), which indicates the point in time when the rate of change in brown bear movement behaviour is the highest; (2) the day when the maximum value of each movement parameter occurs (*max*; for a graphical representation, Fig. 1C), which indicates the point in time when a certain aspect of brown bear movement behaviours are most pronounced. Whereas *k* focuses on the highest rate of change of brown bear movement behaviour at a specific point in time, *max* refers to the maximum movement value at a specific point in time attained by the function as a whole. These two critical points thus provide diverse and valuable information for understanding the dynamics of movement behaviour and how they change according to different factors, like the sex of individuals, for the specific purpose of our study.

We acknowledge that other studies have used other movement metrics such as home range size [31], tortuosity [10], or the mean/median of movement parameters in different seasons [19]. While these metrics are effective for specific study contexts, our selected metrics provide complementary insights. For example, range size captures space use over extended periods but may overlook daily variations that are critical for identifying periods associated with high rates of changes in movement behavioural states. Similarly, tortuosity or linear persistence offers a focused view on directional tendencies but does not quantify the overall extent or intensity of movement. By identifying the timing when the highest rate of change, and the maximum movement value, of brown bear movement behaviour occur, our metrics adds a finer temporal resolution and focuses on capturing potential episodic behaviours that may be obscured in broader measures such as seasonal medians or range sizes.

### Statistical analyses

First, for each movement parameter (i.e., speed, net and total distances), we built a flexible function to describe how adult male and female movement behaviours vary throughout the year. For each movement parameter, we fitted a separate generalised additive mixed model (GAMM) and included the interaction between the day of the year (DOY) and the sex as a smoothing variable using the default thin-plate regression spline in the GAMM4 package in R [45, 46], to allow the relationship to be nonlinear, i.e., the smoothing functions $f\left(v\right)$, $f\left(net\right),$ and $f\left(total\right)$ of male and female brown bears, throughout the year, could potentially take any shape [45]. The goal of these GAMMs models was to test the seasonality of male and female movement behaviours as a first step to then identify our key response variables, i.e. the timing of significant changes in movement dynamics. For doing so, we used the smoothing functions $f\left(v\right)$, $f\left(net\right),$ and $f\left(total\right)$ of male and female brown bears, throughout the year to determine the day of the year (DOY) when brown bears exhibit the highest rate of change (*k*) in their movement parameters, as well as the DOY when they reach their maximum values (*max*). Because movement parameters (i.e., speed, net and total distances) showed skewed and leptokurtic distributions, they were modelled as a Gamma-distributed response variable. When adding the non-linear effects, we always checked the effective degrees of freedom (EDF) of the variables. Those variables showing an EDF < 2 were otherwise included as a linear effect [45, 46]. To account for any potential bias due to differences in the number of observations collected among years and areas, we included the year ID as random factor. Although Slovakian and Romanian bears belong to the same Carpathian population, we treated them as two separate areas in the analyses due to differences in conservation status, management, hunting policies, landscape characteristics, and land use [47]. Finally, we used a Kolmogorov–Smirnov test to additionally compare whether the distribution of the movement parameters (i.e., speed, net and total distances) when these two critical points (i.e., *k* and *max*) occurred was actually different from the distribution of movement values across all days with location fixes.

Second, to study whether, and to what extent, the day when brown bears experience their peak in movement parameters, characterised by the highest rate of change (*k*) and maximum values (*max*) in movement parameters, within the year, depended on the bear sex and country, and after confirming that normality assumptions were met, we further built separate linear mixed models (LMMs) using the lme4 package [48]. We treated *k* and *max* of each movement parameter as normally distributed response variables, with the sex, the country and their interactions as the explanatory variables. As the number of males and females was unbalanced in the different areas, we also included the area and the sex as a nested random factor. Initially, we also included year as a random factor to account for any other potential influential factor varying with year that could otherwise be overlooked. However, due to problems with model convergence, we removed last year as a random factor. All models were evaluated by checking diagnostic plots, and their performance was assessed by estimating *R*-square values. All analyses were performed using R 4.0.4 [49].

## Results

Both male and female brown bears showed a marked seasonal pattern in movement behaviour, as revealed by the GAMMs (Fig. 1A and Table 1). As visually discernible (Fig. 1), whereas male brown bears typically showed a single peak corresponding to both the highest rate of change and the maximum value of speed, net distance and total distance (Fig. 1B), females generally showed two different peaks (Fig. 1C). The differences in the distribution of movement parameters at the day when the highest rate of changes took place (statistically different for speed: *D* = 0.06, *p*-value = 0.005 and total distance: *D* = 0.20, *p*-value = 0.007; but not for *net distance*: *D* = 0.15, *p*-value = 0.17), and at the day when the maximum value of movements occurred (speed: *D* = 0.32, *p*-value < 0.0005; total distance: *D* = 0.53, *p*-value < 0.0005; *net distance*: *D* = 0.20, *p*-value = 0.007), supported the GAMMs results (Table 1) and the visual inspection (Fig. 1) of brown bears showing distinct movement patterns during these two critical points compared to the distribution of movement values across all days with location fixes.

**Table 1:** GAMM coefficients for speed (m/s), net distance (m) and total distance (m) against the smoother term Day of the Year (DOY) for each sex of brown bears studied in in three European areas (Finnish and Russian Karelia, eastern and southern parts of the Romanian Carpathians, and north-central Slovakia) from 2002 to 2021

Dependentvariable		B	SE	*t*-value	*p*-value
Speed (m/s)	Intercept	1.473	0.099	14.83	< 2e-16
		edf		F	*p*-value
	s(DOY): adult females	7.709		56.97	< 2e-16
	s(DOY): adult males	8.368		73.39	< 2e-16
Net distance (m)		B	SE	*t*-value	*p*-value
	Intercept	7.491	0.114	65.87	< 2e-16
		edf		F	*p*-value
	s(DOY): adult females	8.581		15.45	< 2e-16
	s(DOY): adult males	5.851		12.23	< 2e-16
Total distance (m)		B	SE	*t*-value	*p*-value
	Intercept	8.214	0.186	44.16	< 2e-16
		edf		F	*p*-value
	s(DOY): adult females	8.034		25.29	< 2e-16
	s(DOY): adult males	7.608		37.08	< 2e-16

**Fig. 2:**
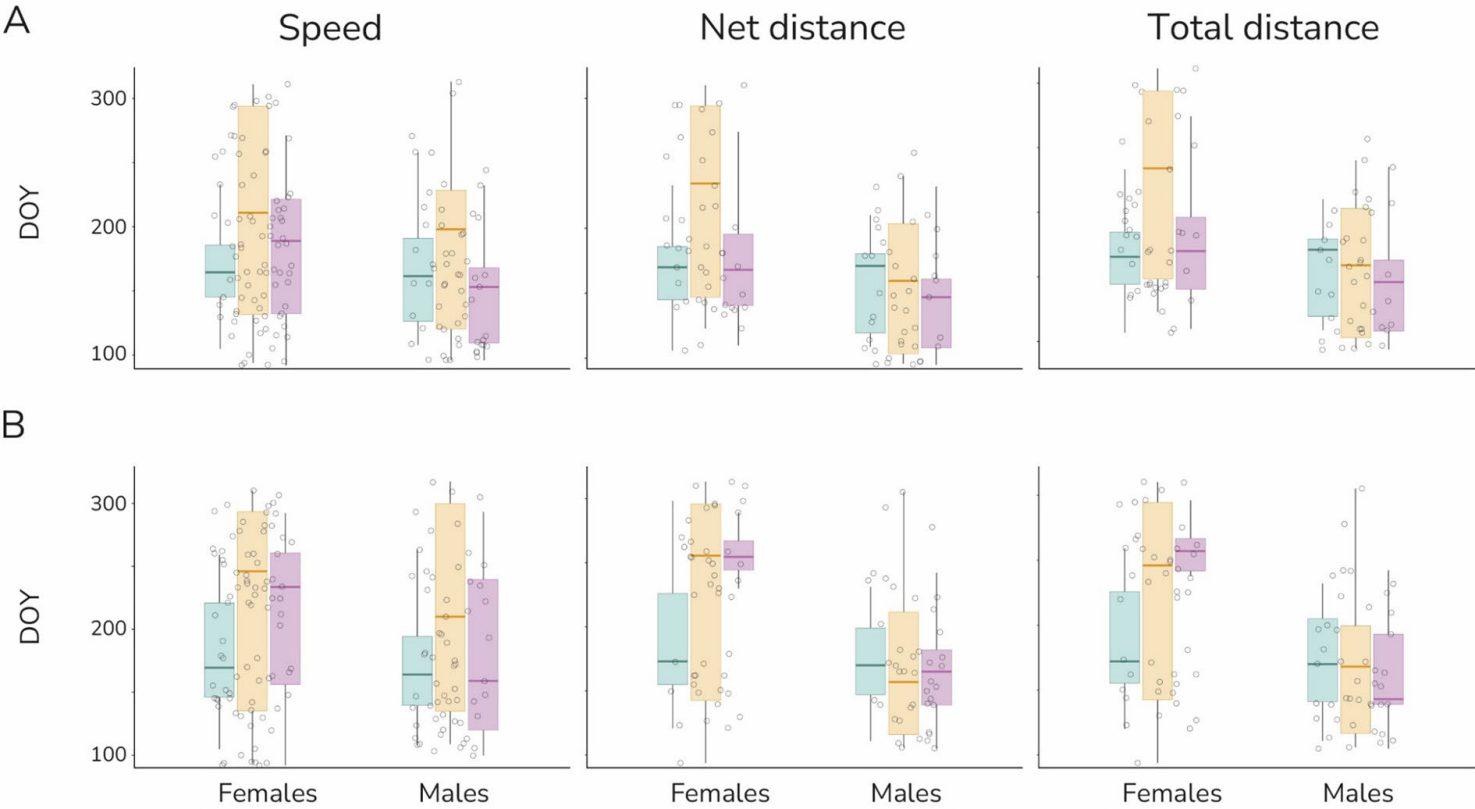
Plots showing the variation of: **A** *k*, the point in time when the rate of change in brown bear movement behaviour was the highest; and **B** *max*, the day when the maximum value of each movement parameter occurred, of each movement parameter across the sex and the areas (light blue = Finnish and Russian Karelia; yellow = eastern and southern parts of the Romanian Carpathians; pink = north-central Slovakia). There were no differences between sexes or areas for *k* and *max*. DOY, day of the year

The day when the highest rate of change in movements took place was not significantly different for males and females (Fig. 2A; Table 2). Both sexes similarly increased their movement activities at the same time of the year (Table 2), corresponding to the mating season. We found a similar pattern for the day when the maximum value of movements took place (Fig. 2B; Table 2, Table 3).

**Table 2:** Model coefficients of linear mixed models (LMMs), treating *k*, i.e. the point in time when the rate of change in brown bear movement behaviour was the highest; and *max*, i.e. the day when the maximum value of each movement parameter occurred of each movement parameter as normally distributed response variables, and the sex, the area, and its interactions, as the explanatory variables

Dependent variable		B	SE	*z*-value	CI	*R*-square
*k* (speed)	Intercept	172.42	32.38	5.24	[149.84,194.98]	0.55
	Romania	52.43	46.13	1.12	[18.81,86.08]	
	Slovakia	0.50	46.43	0.01	[− 34.69,35.70]	
	Adult males	− 16.49	45.32	0.36	[− 49.59,16.63]	
	Romania: adult males	− 48.71	66.19	0.72	[− 105.24,7.82]	
	Slovakia: adult males	− 10.01	64.22	0.15	[− 57.36,37.34]	
*k* (net distance)	Intercept	168.55	29.47	5.66	[147.41,189.661	0.39
	Romania	43.78	41.82	1.00	[13.45,74.13]	
	Slovakia	10.85	41.41	0.26	[− 17.41,39.14]	
	Adult males	− 5.56	33.53	0.16	[− 36.72,25.64]	
	Romania: adult males	− 17.39	49.31	0.35	[− 68.61, 33.82]	
	Slovakia: adult males	− 20.61	46.94	0.43	[− 62.55, 21.32]	
*k* (total distance)	Intercept	170.84	49.53	3.40	[148.84,192.83]	0.55
	Romania	54.08	70.32	0.76	[20.83,87.36]	
	Slovakia	1.83	70.52	0.03	[− 33.01,36.681	
	Adult males	− 15.44	44.66	0.34	[− 48.17,17.30]	
	Romania: adult males	− 50.30	65.34	0.76	[− 106.71, 6.08]	
	Slovakia: adult males	− 10.04	63.33	0.16	[− 57.18,37.091	
*Max* (speed)	Intercept	192.42	34.52	5.48	[166.08,218.74]	0.36
	Romania	36.29	49.24	0.72	[− 2.94,75.54]	
	Slovakia	49.16	49.64	0.97	[8.11,90.23]	
	Adult males	− 21.89	45.73	0.47	[− 60.51,16.75]	
	Romania: adult males	− 30.67	67.47	0.45	[− 96.61, 35.28]	
	Slovakia: adult males	− 46.75	64.83	0.71	[− 101.99,8.48]	
*Max* (net distance)	Intercept	182.35	28.30	6.38	[158.76,205.95]	0.21
	Romania	36.15	40.18	0.89	[2.28,70.21]	
	Slovakia	28.42	39.67	0.71	[− 3.13,60.00]	
	Adult males	− 11.31	33.52	0.33	[− 46.11,23.51]	
	Romania: adult males	6.99	49.70	0.14	[− 50.18, 64.18]	
	Slovakia: adult males	− 22.71	46.76	0.48	[− 69.56, 24.08]	
*Max* (total distance)	Intercept	183.28	18.82	9.79	[158.77,205.92]	0.21
	Romania	35.36	27.48	1.27	[2.28,70.20]	
	Slovakia	54.71	28.12	1.91	[− 3.13,60.00]	
	Adult males	− 15.96	21.71	0.72	[− 46.11,23.50]	
	Romania: adult males	− 30.70	35.86	0.84	[− 50.18, 64.18]	
	Slovakia: adult males	− 60.58	31.11	1.92	[− 69.56, 24.08]	

The reference categories for sex and area are females and the Finnish and Russian Karelia, respectively. Confidence intervals not overlapping zero represent a significant effect of each explanatory variable

**Table 3:** Descriptive statistics (*mean* ± *SD; range*) by: (a) sex for the day when the rate of change in brown bear movement behaviour was the highest (*k*); and (b) for the day when the maximum value of each movement parameter occurred (*max*)

		Speed (mean ± SD; range)	Net distance (mean ± SD; range)	Total distance (mean ± SD; range)
*k*	Males	152 ± 45 days; 95–258 days	163 ± 55 days; 96–313 days	152 ± 45 days; 94–257 days
	Females	190 ± 56 days; 106–310 days	186 ± 59 days; 92–311 days	193 ± 57 days; 107–311 days
*Max*	Males	173 ± 51 days; 105–305 days	181 ± 62 days; 100–317 days	169 ± 49 days; 105–305 days
	Females	218 ± 64 days; range = 94–313 days	204 ± 64 days; 92–310 days	213 ± 61 days; 94–310 days

Additionally, although the day when the highest rate of change in brown bear movement behaviour was similar across all three brown bear populations (Fig. 2A; Table 2), it tended to correspond with the abiotic gradient among the areas, with brown bears showing the highest rate of change later at the southernmost sites. Moreover, the maximum value for Finnish brown bears occurred earlier in the year, generally aligning with the mating season (Fig. 2B; Table 2). In contrast, Romanian and Slovakian brown bear females travelled the maximum net and total distances later in the year, during the hyperphagia period, compared to males (Fig. 2B). Overall, these results suggest that local conditions also influenced the day when both the highest rate of change and the maximum value of the movement parameters occurred.

## Discussion

When exploring the movement patterns of adult female brown bears within the year, we have identified that, as it is the case for males, one of the highest rates of change in the female movement patterns occurred during the mating season, suggesting that adult females may play a more active role in searching for males than previously thought. This indicates that it may not necessarily be true that brown bear males are the only (or prevailing) roaming-to-mate sex. Indeed, the highlighted patterns of bear movements, with males showing a peak in speed and distance during the mating season while females exhibit a peak in travelled distances, suggest that females may actively seek out males over longer distances during the mating season.

Our result is in line with previous studies demonstrating that estrous female brown bears show larger home ranges during the mating season than in the post-mating season [50], which might be partially due to female roaming to enhance opportunities to encounter prospective mates. In this regard, the use of multi-country data, specifically data from radio-collared males and females in three distinct areas (Finland, Slovakia, and Romania), enhances the strength of our analyses by enabling us to assess the potential generalisability of our results across different populations.

Our results challenge the common view that only one sex tends to dominate the direction of evolution of given reproductive strategies in particular taxa [6]. From the perspective of mating competition, the influence of female competition in the sexual selection of mammals becomes apparent when females compete for the sperm of favoured or competitively successful males [3]. Females are mostly the more selective sex in mate selection, due to their higher reproductive investment than males [8], thus active mate searching for the best mates is also expected. Because brown bear females seem to be choosy during mating, i.e., preferably select for high-quality males [37, 51, 52], longer displacement may increase the likelihood of encountering best mates. Similarly to female brown bears in our study, large herbivore females tend to exhibit increased mobility during the breeding season. For instance: (a) movements beyond their normal range are commonly observed among roe deer *Capreolus capreolus* females during the reproductive period [53–55], potentially serving as an alternative strategy to avoid mating with closely related males [53]; and (b) female white-tailed deer *Odocoileus virginianus* appear to maximize the quality of their mates by increasing their movement rates near the peak of the breeding season [56].

Additionally, selection pressures may also arise from female intrasexual competition to acquire additional advantages, resulting in diverse competitive strategies. In the case of brown bears, females face the threat of infanticide, a primary source of cub mortality, with males typically unrelated to the cubs they kill [21–23, 30]. To mitigate the likelihood of infanticide, females usually copulate with multiple males during each mating season. This behaviour increases the likelihood that, upon den emergence with cubs the following year, females will encounter potential male parents again. Such encounters serve as a deterrent to infanticide, as males are less likely to harm cubs that could be their offspring [21, 22]. It is thus not surprising that one of the periods of the largest adult female movements is the mating period. Actively searching for males, rather than passively waiting for them, is what we would expect from a species whose primary strategy to avoid infanticide is to engage in copulations with as many different males as possible during each mating season.

The highlighted increase in female movements during the mating season aligns well with the encounter theory regarding potential mates [35], with bears increasing their displacement to cover larger areas during the mating period [19]. Considering that the females’ impulse to reproduce, shared with males, is also accompanied by the need to avoid the possibility that their cubs will suffer infanticide, it might not be surprising that some females will roam more actively than males during mate searching. This is especially true under certain ecological conditions, such as the density and movements of adult males. This possibility is not only supported by our results but also, at least indirectly, by evidence that males can locally exhibit larger home ranges during the hyperphagia period than during the mating season [21, 22, 57]. An additional, not mutually exclusive explanation for movement patterns may be that increased adult female movements during mate searching are influenced by the proximity to the nearest neighbouring female [58]. This is due to the negative effect of distance to the nearest neighbour on female reproductive success, known as female-induced reproductive suppression [58–60].

Because animal reproductive strategies are shaped by competing interests, with opportunities and constraints dictated by the environment [6], movement patterns can also reflect local influences. These influences are likely determined by local conditions such as abiotic gradients among the study sites [61–63], as well as local feeding strategies and types of diet [64]. Thus, local differences in female movement patterns represented in Fig. 2 may be attributed to the context-dependent nature of female choice [51]. For example, female movements could be influenced by factors such as density, availability and distribution of males, particularly of high-quality mates [51], as well as food distribution and availability, particularly during hyperphagia. This may help explaining why Slovakian females travelled the greatest net and total distances during the hyperphagia period. In contrast, the less variable movement patterns observed in Finnish bears might result from the abundance of artificial feeding points in autumn [41]. The effect of local factors on movements during mate searching, which may reflect local adaptation to given conditions, has also been described for brown bears inhabiting a marginal Arctic landscape. In such environments, it has been suggested that finding quality habitats that help to facilitate greater reproductive success takes precedence over mate searching [19].

Serious challenges to our understanding of reproductive strategies and systems have been posed since the nineties, including appreciation of an active role played by females in many taxa, in which females have control over mating opportunities and copulations, and may have options for controlling paternity, e.g., by manipulating the timing of mating or by ‘postcopulatory choice’ [6]. Our results support this need for more detailed investigation to determine the mechanisms and evolutionary consequences of female behaviours across a broad range of mammalian taxa [3]. A more active role of females during mate searching, as well as competition between them, may potentially be an important selection pressure in the evolution of mammalian reproductive strategies [3]. The local differences highlighted here also underscore the importance of further studies to determine how social and ecological conditions explain variation in the form and intensity of the female role in mate searching [3]. Future research in this field offers stimulating potential to advance our current understanding of mammalian social and mating systems.

## Supplementary Information

Supplementary file 1.

## Data Availability

The dataset is available in the Supplementary Information.

## Abbreviations

PDOP: Positional dilution of precision
v: Movement speed
tot: Total distance
net: Net distance
k: The day when the maximum of the first derivative of each movement parameter takes place
max: The day when the maximum value of each movement parameter occurs
GAMMs: Generalised additive mixed models
DOY: Day of the year
LMMs: Linear mixed models
